# Simplexidine, a 4-Alkylpyridinium Alkaloid from the Caribbean Sponge *Plakortis simplex*

**DOI:** 10.3390/molecules13071465

**Published:** 2008-07-17

**Authors:** Ernesto Fattorusso, Adriana Romano, Fernando Scala, Orazio Taglialatela-Scafati

**Affiliations:** Dipartimento di Chimica delle Sostanze Naturali, Università di Napoli “Federico II”, Via D. Montesano 49, 80131 Napoli, Italy.; E-mails: fattoru@nina.it; adromano@unina.it; fernando.scala@unina.it

**Keywords:** Simplexidin, alkaloids, pyridinium, *Plakortis*, NMR

## Abstract

Chemical analysis of the secondary metabolites of the Caribbean sponge *Plakortis simplex*, a source of many bioactive compounds, showed the presence of the new metabolite simplexidine (**4**), belonging to the extremely rare class of 4-alkyl-pyridinium alkaloids. The structural characterization of this molecule, based on spectroscopic methods, is reported.

## Introduction

As part of an ongoing search for biologically active compounds from marine invertebrates, our research group has been devoting considerable efforts to the chemical investigation of the Caribbean sponge *Plakortis simplex*. This research project, started about ten years ago, has been strongly fostered by the discovery of a large array of structurally unique and biologically active metabolites. This richness of secondary metabolite production has been discussed in a review paper [1] and has been recently correlated to the wide presence of bacterial and fungal symbionts associated to the sponge host cells [2].

**Figure 1:**
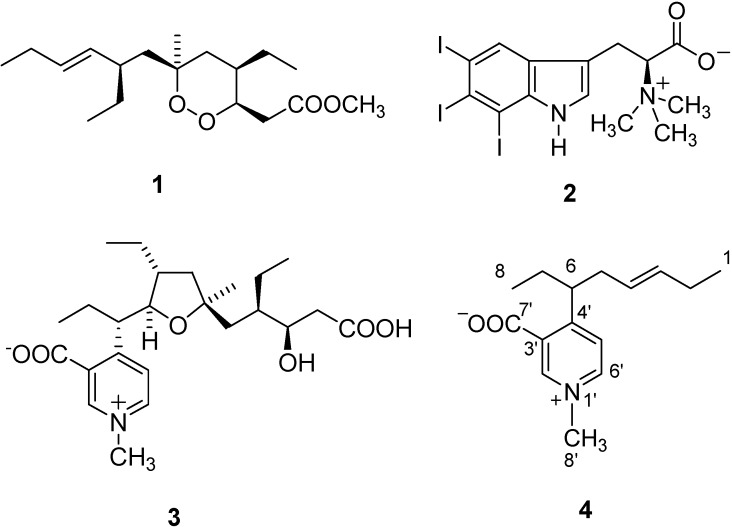
Representative molecules from *Plakortis simplex*, including the new simplexidine (**4**).

For example, *P. simplex* provided a series of promising lead compounds for drug development, including the immunosuppressive glycolipids plakosides [3] and simplexides [4] and a series of endoperoxide-containing derivatives related to plakortin (**1**, Figure 1) [5,6], for which a potent antimalarial activity (in the nM range) has been discovered [7].

Structurally unique metabolites were also disclosed as components of the most polar fractions obtained from the organic extracts of *P. simplex*. Indeed, these fractions showed to contain the first natural betaine derivatives to be characterized by a iodinated indole ring [8,9], e.g. plakohypaphorine E (**2**, Figure 1), and the unique 4-alkylpyridinium alkaloid simplakidine (**3**, Figure 1) [10]. Careful examination of the polar fractions of *P. simplex* has now yielded to the isolation of a new 4-alkyl-pyridinium alkaloid that we have named simplexidine (**4**, Figure 1) and for which we report herein details about isolation and structural elucidation.

## Results and Discussion

A specimen of the sponge *Plakortis simplex* (Demospongiae, order Homosclerophorida, family Plakinidae) was collected during the summer of 2002 along the coasts of The Bahamas and immediately frozen. After homogenization, the organism was exhaustively extracted, in sequence, with methanol and chloroform. The methanolic layer was partitioned between *n*-BuOH and water, and, subsequently, the organic phase, combined with the CHCl_3_ extract, was subjected to chromatography over a reversed phase (RP_18_) silica column eluted with a solvent gradient from H_2_O/MeOH 9:1 to MeOH and then to MeOH/CHCl_3_ 9:1. The most polar fractions were preliminarily separated over silica gel (gradient from EtOAc to MeOH) and then re-chromatographed by reverse-phase HPLC (eluent MeOH/H_2_O 4:6) to finally yield 2.0 mg of pure simplexidine (**4**).

The molecular formula C_15_H_21_NO_2_ was assigned to simplexidine ([α]_D_ = –5.8) based on mass spectrometry evidence [ESI-MS (negative ions): *m/z* 246 (M-H)^-^; ESI-MS (positive ions): *m/z* 248 (M+H)^+^, 270 (M+Na)^+^; HR-FABMS: *m/z* 248.1647 (M+H)^+^, calcd. for C_15_H_22_NO_2_ *m/z* 248.1651].

The ^1^H-NMR spectrum of simplexidine (Table 1) showed three signals (a singlet at δ_H_ 8.81 and two doublets at δ_H_ 8.63 and 7.91) in the aromatic region, three methine multiplets (δ_H_ 5.48, 5.35, and 3.81) and a methyl singlet (δ_H_ 4.34) in the midfield region, and a series of well resolved signals confined in the spectral region ranging from δ_H_ 2.50 to 0.80, including two methyl triplets at δ_H_ 0.93 and 0.89. The presence of an aromatic chromophore in the structure of simplexidine (**4**) was further suggested by the UV absorption at λ_max_ 268 nm and by the presence of five signals between δ_C_ 127 and 164 in the ^13^C-NMR spectrum (Table 1).

The 15 carbon signals present in the ^13^C-NMR spectrum were assigned, with the aid of DEPT experiments, to three methyls, three methylenes, six methines and three unprotonated sp^2^ type carbons. Among these latter resonances, the signal at δ_C_ 168.6 could be ascribed to a carboxylate group, as suggested also by the IR absorption at ν_max_1642 cm^-1^. Association of the resonances of the 12 proton-bearing carbons with those of the relevant protons was accomplished through the analysis of a 2D NMR gradient-HSQC spectrum.

**Table 1:** ^1^H (500 MHz) and ^13^C (125 MHz) NMR data of simplexidine (**4**) in CD_3_OD.

Pos.	δH, mult., *J* in Hz	δC, mult.	Pos.	δH, mult., *J* in Hz	δC, mult.
1	0.93, t, 7.3	14.5, CH_3_			
2	1.97, q, 7.3	25.8, CH_2_	2’	8.81, s	143.1, CH
3	5.48, dt, 15.4, 7.3	136.2, CH	3’		141.9, C
4	5.35, dt, 15.4, 7.0	126.2, CH	4’		163.5, C
5	2.43, q, 7.0	38.1, CH_2_	5’	7.91, d, 6.9	127.3, CH
6	3.81, m	43.4, CH	6’	8.63, d, 6.9	143.4, CH
7a	1.87, m	27.7, CH_2_	7’		168.6, C
7b	1.73, m		8’	4.34, s	46.7, CH_3_
8	0.89, t, 7.5	12.2, CH_3_			

**Figure 2:**
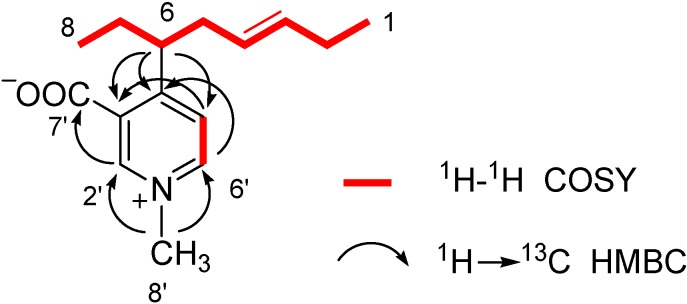
^1^H-^1^H COSY and key ^2,3^*J*_C-H_ HMBC correlations of simplexidine (**4**).

Inspection of the ^1^H-^1^H COSY NMR spectrum of simplexidine (**4**) allowed us to arrange all the proton multiplets within the two spin systems showed in red in Figure 2. The first fragment includes only the two mutually coupled aromatic doublets at δ_H_ 8.63 and 7.91, while the second moiety is an eight-carbon fragment connecting the two methyl triplets (from H_3_-1 to H_3_-8) and comprising two coupled sp^2^ methines (H-3 and H-4) and a single branching at the sp^3^ methine C-6 (δ_H_ 3.81).

With these data in our hands and taking into account the molecular formula, the assembly of the carbon framework of simplexidine (**4**) required the elucidation of an aromatic C_7_H_6_NO_2_ subunit, probably linked at C-6 and comprising a carboxylate group. Interpretation of diagnostic gradient-HMBC cross-peaks (Figure 2) was of pivotal importance to resolve this issue and suggested the presence of a disubstituted *N*-methyl pyridinium ring. This assignment was corroborated by perfect agreement of ^1^H- and ^13^C-NMR resonances of this subunit with literature values [10,11].

In particular, the *N*-methyl singlet at δ 4.34 (H_3_-8’) showed g-HMBC cross-peaks with two almost overlapped protonated carbons at δ_C_ 143.1 (C-2’, H-2’ = δ_H_ 8.81, s) and 143.4 (C-6’, H-6’ = δ_H_ 8.63, d, *J* = 6.9 Hz). Substitution at C-2’ and C-6’ of the pyridinium ring was consequently excluded, and, since considering the doublet nature of H-6’, C-5’ must be protonated as well, this leaves only a 3,4 disubstitution as possible. The intense g-HMBC cross-peak H-2’/C-7’ suggested the placement of the carboxylate group at C-3’, while the cross peaks H-6/C-3’, H-6/C-4’, H-6/C-5’ and H-5’/C-6 confirmed the linkage of the alkyl chain at C-4’, thus completely defining the planar structure of simplexidine (**4**). The coupling constant *J*_H-3/H-4_ = 15.4 Hz was indicative of the *trans* geometry of the Δ^3,4^ double bond, while, also considering the limited amounts of sample available (2.0 mg), strategies aimed at the definition of the absolute configuration at the single stereogenic carbon C-6 were not undertaken.

**Figure 3:**
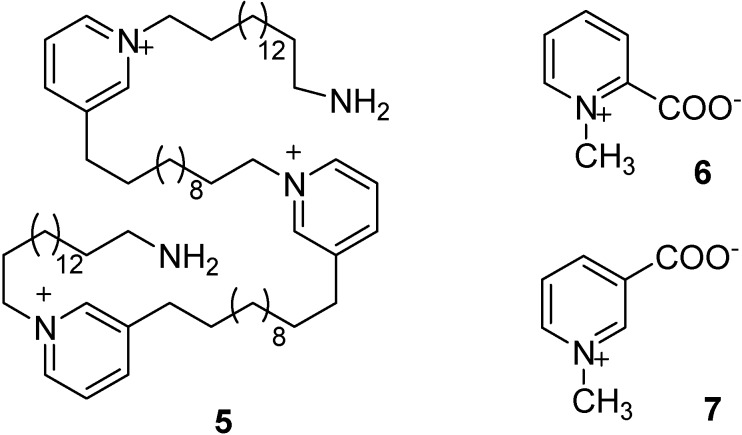
Chemical structures of pachychaline C (**5**), homarine (**6**) and trigonelline (**7**).

Pyridinium alkaloids are frequently isolated from the polar extracts of marine invertebrates, mostly sponges; however, in spite of the wide diffusion, the chemical diversity within this class of compounds is somewhat limited and only two structural groups can be identified. The most common group of pyridinium derivatives includes oligomeric structures with alkyl linear chains linked at positions C-3 and N-1 of the pyridinium ring, e.g. the recently reported pachychaline C (**5**, Figure 3) [12]. These molecules are known to exhibit a range of bioactivities including cytotoxic [13] and anti-cholinesterase [14] properties. The second structural group includes the carboxyl-containing homarine (**6**, Figure 3) or trigonelline (**7**, Figure 3). substituted at C-3 or C-2, respectively, with short alkyl chains.

The isolation of simplexidine (**4**) is particularly remarkable since it confirms the existence, within the *Plakortis simplex* biosynthetic machinery, of enzymes deputed to the attachment of polyketide-derived carbon chains at the 4 position of pyridinium rings. In the case of simplakidine (**3**), this unique reaction led to the linkage between the trigonelline nucleus and a C_17_ moiety that clearly shared the plakortin (**1**) carbon backbone. On the other hand, the biosynthetic origin of the C_8_ group present in the structure of simplexidine (**4**) cannot be unambiguously predicted, although similarly to that postulated for plakortin derivatives [15], a polyketide origin can be hypothesized also in this case. Investigation of the role of microbial symbionts in the elaboration of the incredible pool of structurally unique secondary metabolites of *Plakortis simplex* is in progress in our lab. Simplexidine (**4**) exhibited very weak cytotoxicity toward murine macrophages (RAW 264-7) with 30% of growth inhibition at 80 μg/mL.

## Experimental

### General

Optical rotations were measured in MeOH on a Perkin-Elmer 192 polarimeter equipped with a sodium lamp (λ = 589 nm) and a 10-cm microcell. IR (KBr) spectra were recorded on a Bruker model IFS-48 spectrophotometer. UV spectra were obtained in MeOH using a Beckman DU70 spectrophotometer. ESI-MS spectra were performed on a LCQ Finnigan MAT spectrometer. HR-FABMS were performed on a FISONS Prospec mass spectrometer using a glycerol matrix. ^1^H (500 MHz) and ^13^C (125 MHz) NMR spectra were measured on a Varian INOVA 500 spectrometer; chemical shifts are referenced to the residual solvent signal (CD_3_OD: δ_H_ = 3.34, δ_C_ = 49.0). The multiplicities of ^13^C resonances were determined by DEPT experiments. Homonuclear ^1^H connectivities were determined by using COSY experiments. One bond heteronuclear ^1^H-^13^C connectivities were determined with gradient-HSQC pulse sequence. Two- and three-bond ^1^H-^13^C connectivities were determined by gradient-selected HMBC experiments optimized for a ^2,3^*J* of 7.0 Hz. Medium-pressure liquid chromatographies (MPLC) were performed using a Büchi 861 apparatus with RP_18_ and SiO_2_ (230-400 mesh) stationary phases. High performance liquid chromatography (HPLC) separations were achieved in isocratic mode on a Beckmann apparatus equipped with RI detector and LUNA (Phenomenex) columns (SI60, 250 × 4 mm).

### Extraction and Isolation Procedure

A specimen of *Plakortis simplex* was collected in July 2002 along the coasts of The Bahamas. A voucher specimen is deposited at the Dipartimento di Chimica delle Sostanze Naturali, Italy with the ref. n° 02-10. The organism was immediately frozen after collection and kept frozen until extraction, when the sponge (43 g, dry weight after extraction) was homogenized and extracted with methanol (4 × 500 mL) and with chloroform (4 × 500 mL). The methanol extract was initially partitioned between H_2_O and *n*-BuOH and then the organic phase was combined with the CHCl_3_ extract and concentrated *in vacuo* to afford a brown oil (22.1 g). This was subjected to chromatography on a column packed with RP_18_ silica gel and eluted with 9:1 H_2_O/MeOH (A_1_), 7:3 H_2_O/MeOH (A_2_), 4:6 H_2_O/MeOH (A_3_), 2:8 H_2_O/MeOH (A_4_), MeOH (A_5_), and 9:1 MeOH/CHCl_3_ (A_6_). Fraction A_3_ (442 mg) was further chromatographed by MPLC (SiO_2_ 230-400 mesh; solvent gradient system of increasing polarity from EtOAc to MeOH). Fractions eluted with EtOAc/MeOH 2:8 were re-chromatographed by reverse-phase HPLC (eluent MeOH/H_2_O 4:6) affording pure simplexidine (**4**, 2.0 mg).

### Simplexidine (**4**) Characterization Data

Colorless amorphous solid. [α]_D_^25^ –5.8 (c = 2.0 mg/mL in MeOH); IR (KBr): ν_max_ 1642, 1078, 922 cm^-1^; UV (MeOH): λ_max_ 268 (logε 3.41); ESI-MS (negative ions): *m/z* 246 (M-H)^-^. ESI-MS (positive ions): *m/z* 248 (M+H)^+^, 270 (M+Na)^+^. HR-FABMS analysis: *m/z* 248.1647 (M+H)^+^, calcd. for C_15_H_22_NO_2_ *m/z* 248.1651. ^1^H- and ^13^C-NMR: Table 1.
